# Portable low-cost open-source wireless spectrophotometer for fast and reliable measurements

**DOI:** 10.1016/j.ohx.2020.e00108

**Published:** 2020-04-18

**Authors:** Katrina Laganovska, Aleksejs Zolotarjovs, Mercedes Vázquez, Kirsty Mc Donnell, Janis Liepins, Hadar Ben-Yoav, Varis Karitans, Krisjanis Smits

**Affiliations:** a Institute of Solid State Physics, University of Latvia, Kengaraga Str. 8, Riga LV-1063, Latvia; b School of Chemical Sciences, National Centre for Sensor Research, Dublin City University, Glasnevin, Dublin 9, Ireland; c Department of Biomedical Engineering and Ilse Katz Institute of Nanoscale Science and Technology, Ben-Gurion University of the Negev, Beer Sheva 8410501, Israel; d Institute of Microbiology and Biotechnology, University of Latvia, Jelgavas Str. 1, Riga LV-1004, Latvia

**Keywords:** Absorption, Spectroscopy, Mobile application, Arduino, Spectrometer

## Abstract

We demonstrate a low-cost standalone portable spectrophotometer for fast and reliable measurement execution. The data acquired can be both displayed via a dedicated smartphone application or a computer interface, allowing users either to gather and view data on the move or set up a continuous experiment. All design and software files are open-source and are intended for the device to be easily replicable and further customizable to suit specific applications. The assembled device can measure absorption in the wavelength range from 450 nm to 750 nm with a resolution of 15 nm and is housed in a 90 × 85 × 58 mm casing. Validation of the device was carried out by assessing wavelength accuracy, dynamic range and the signal-to-noise ratio of the system, followed by testing in three different applications where limit of quantification, limit of detection and relative standard deviations were determined. The results indicated better performance than low-cost spectrophotometers, on average being comparable to moderate to high-cost spectrophotometers.

## Graphical abstract

**Figure:**
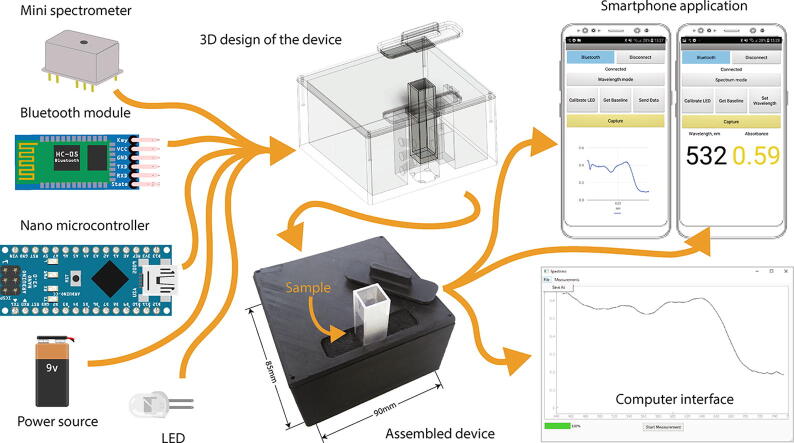

**Specifications table:**

**Table:** 

Hardware name	Open-Source Miniature Spectrophotometer
Subject area	General
Hardware type	• Measuring physical properties and in-lab sensors
	• Field measurements and sensors
Cost of hardware	225EUR
Source file repository	All supporting software and descriptions are available at https://doi.org/10.17605/OSF.IO/RBFSE.

## 1 Hardware in context

Spectrophotometric systems are widely used in studies across many fields such as physics, materials science, chemistry, biochemistry, biomolecular chemistry, photovoltaics and more [7], [3], [18], [2], [17], [14], [8]. Many of these applications require highly precise and reliable data which often leads to the implementation of complex and expensive spectrophotometric systems. However, in addition to rendering impossible the range of applications which require portability or good time resolution (measuring the whole spectrum at once as opposed to scanning), when performing simpler applications such as day-to-day measurements or pilot studies, convenience can often outweigh the need for precision, making the complexity of classical systems an unnecessary and a time consuming obstacle.

There have been several projects that have focused on building a portable and compact spectrophotometer that could be used on-the-go [20], [1], [6], [21], [4], however, most of these projects have been aimed at creating very low-cost devices, usually to be used for educational purposes [1], [6], [21] or to obtain simple and limited information and not for acquiring scientifically significant data, which requires reproducible and accurate measurements.

Commercially available handheld mini-spectrophotometers on the other hand are mostly used in a reflective configuration [5], [15] and obtain qualitative information about the samples. Although analyzing the reflected light has its advantages (no sample preparation, can measure solids etc.), it can not offer the quantitative results that are needed to acquire absolute values. This is due to the spectrometer not collecting all of the reflected light, as is achieved with the transmitted light in the transmission configuration.

In this work we describe the construction and performance of the portable, low-cost, customizable and fast (registers the whole wavelength range at once, allowing to obtain a full absorbance spectrum every second) open-source miniature spectrophotometer (OSMS).

## 2 Hardware description

The OSMS device is based on the Hamamatsu C12880MA spectrometer chip, used in a transmission configuration. Transmittance configuration was chosen over reflectance as it offers absolute values instead of relative values that are given by reflectance. The chip is controlled by simple and widely-available microcontrollers and data is transferred via Bluetooth to a mobile application. Connection to a PC via USB interface is also possible for more complex (absorbance-over-time, data accumulation etc.) data acquisition and analysis (e.g. kinetics measurements). The device can measure absorption in the range from 450 nm to 750 nm and uses a single warm white LED as the light source. The open-source miniature spectrophotometer (OSMS) proposed in this study can be adapted to suit specific fields such as food analysis, water quality, biological research, clinical diagnostics and others by adjusting the software or adding additional LED light sources.

**Fig. 1:**
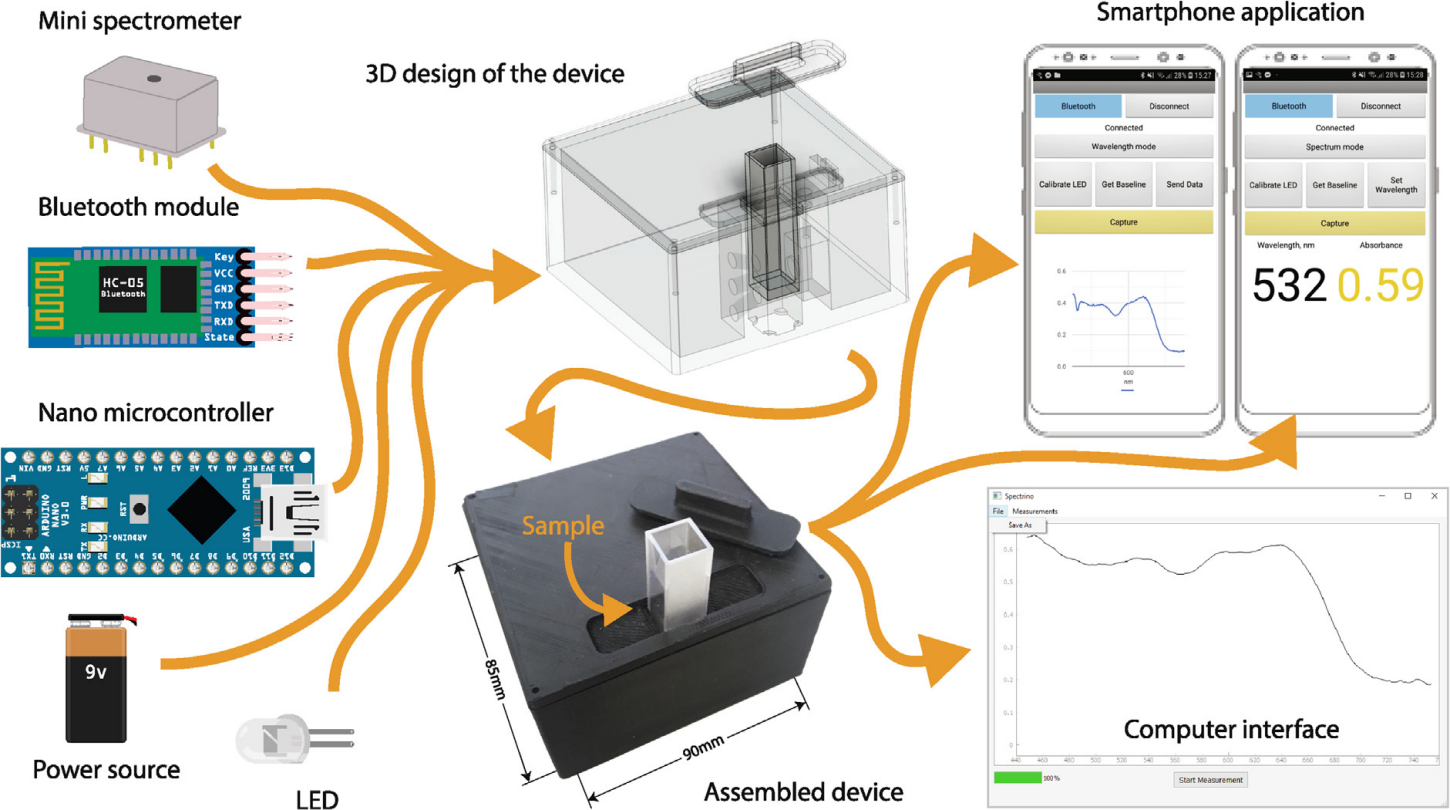
The main components of the device – the mini-spectrometer, a Bluetooth module, nano microcontroller, an LED and a power source are featured on the left side. The 3D design and the assembled device can be seen in the middle of the figure and the smartphone application and the computer interface are on the right.

The miniature spectrophotometer device consists of only 4 main components – an LED, a spectrometer, a microcontroller and a bluetooth module (and a power source if used in portable mode) as can be seen in Fig. 1 and allows the user to acquire absolute absorbance values of the measured samples. The case accommodates for the insertion of a standard cuvette (10 mm × 10 mm) which contains the sample to be measured. Although the device is generally designed for liquid samples, solid samples can also be measured if attached to a quartz holder.

• The recorded data can be easily viewed and sent to a computer using the mobile application and be analyzed from thereon.
• The device registers the full spectrum at once, making it especially attractive to studies that are time sensitive.
• The size of the finished device is fitting for field applications where larger equipment would be difficult to use.

A note on why the visible range was chosen: the Hamamatsu miniature spectrometer chips are only available for visible range. Spectrometers for other ranges are significantly costlier (up to 20×) and also larger in size. The second reason is the light source. Nichia Optisolis was chosen as it emits in a wider range of spectrum than a regular white LED and we suggest others who build the device to choose similar LEDs. However, even with improved range of spectrum, the LED does not cover wavelengths over 780 nm. 750 nm was chosen as the limit in our device, even though the spectrometer chip itself can cover up to 850 nm (for the same reason we chose a lower limit of 450 nm, even though the limit of the spectrometer itself is 340 nm. As was already mentioned it is possible to add additional LEDs to cover other specific ranges.

## 3 Design files

### 3.1 Electronics

A schematic of the components was shown in Fig. 1 The device consists of the Hamamatsu C12880MA spectrometer, a white LED (Nichia Optisolis was used), an Arduino Nano microcontroller for control and readout of data, and a Bluetooth module for data transfer to the mobile application. The case of the spectrophotometer was 3D printed and a 10 mm × 10 mm (inner dimensions) cuvette is to be used for the samples. After the sample measurement is obtained, the calculated absorption spectrum is sent to the smartphone via the custom mobile application and the data is displayed there. From the application, the data then can be forwarded via e-mail for further analysis.

**Fig. 2:**
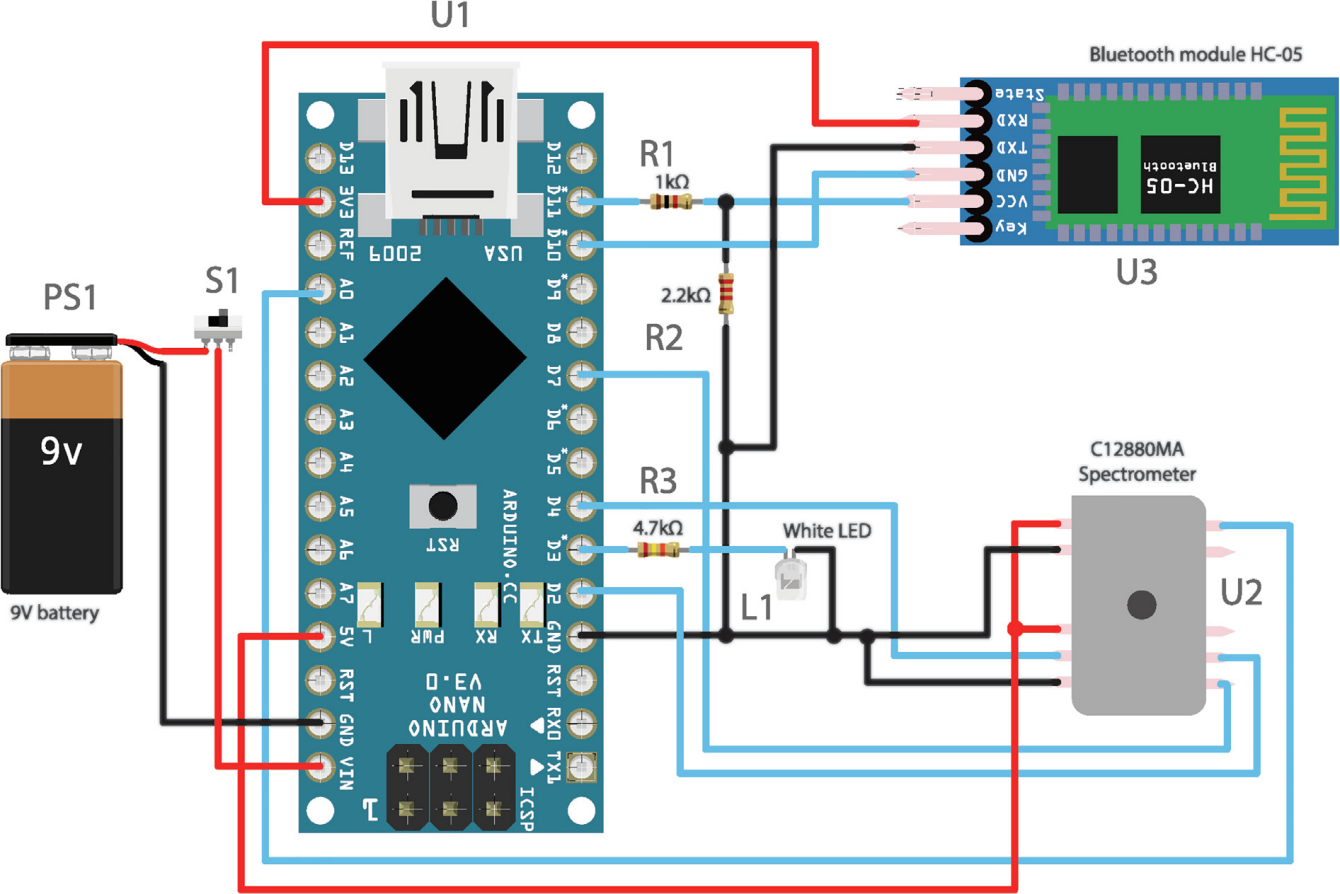
The schematic drawing of the spectrophotometer.

The schematic in Fig. 2 shows the wiring between all elements. There are three main components: C12880MA spectrometer chip, HC-05 Bluetooth module, and an Arduino Nano.

The Hamamatsu C12880MA spectrometer chip is connected to Arduino Nano both for voltage supply and for readouts. Pins 4, 6 and 7 are Clock, Start Pulse, and Trigger pulse and are connected to the digital output pins of the Arduino and the Video signal on pin 10 is read via the analog input pin A0.

The resistors R_1_ and R_2_ work as a voltage divider, transforming the 5 V signals coming from Arduino to 3.3 V which are needed according to the HC-05 module datasheet. The TX pin is left unmodified as the Arduino TTL levels will register a 3.3 V as a high state. The TX and RX of the HC-05 is connected to the digital pins D_11_ and D_10_ of Arduino, that are set up to act as a software serial port. Arduino itself is attached to a 9 V power source via a simple switch.

### 3.2 3D printing

**Fig. 3:**
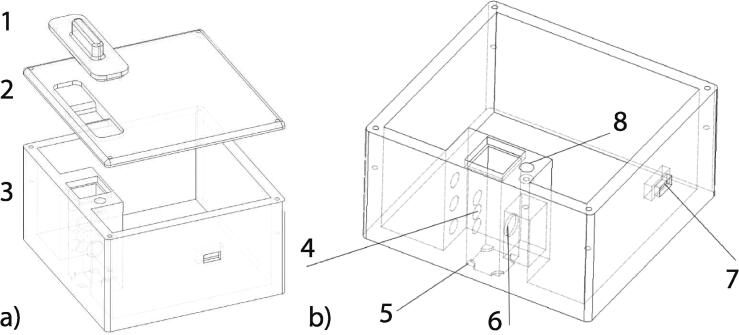
a) overview of the construction. 1 top cap for filtering out the ambient light, 2 Screw-in lid, 3 main body containing all elements; b) overview of the main body. 4 mounting spots for LEDs, 5 stop features for the cuvette, 6 opening and mounting point for C12880MA unit, 7 on/off switch mounting point, 8 fixing screw hole for C12880MA unit.

**Fig. 4:**
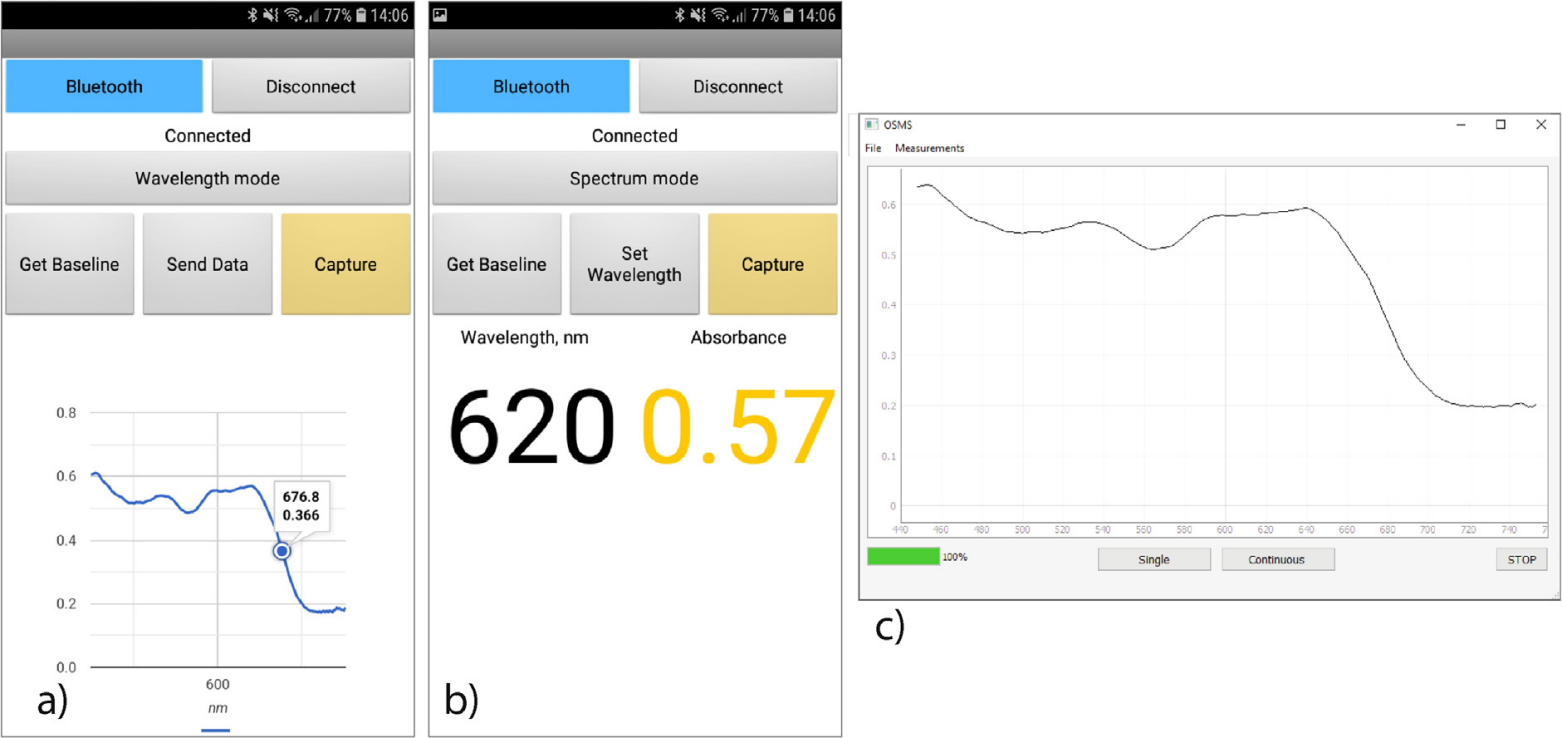
a) Android application in full absorbance spectrum mode; b) Android application in single wavelenght mode; c) Python application for connection via USB.

**Fig. 5:**
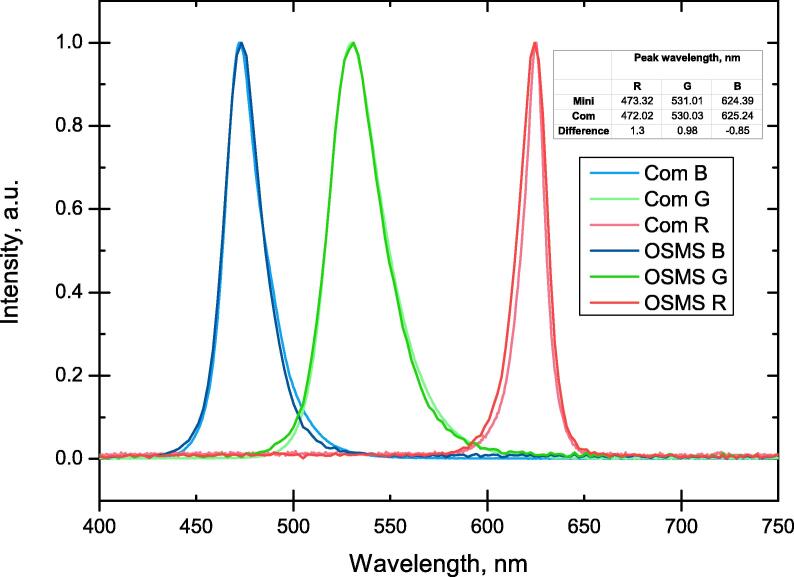
Spectra of an RGB LED with each color component turned on separately. Inset table shows the position of maxima and the difference when measured by the miniature spectrophotometer OSMS and the commercial system *Com*.

**Fig. 6:**
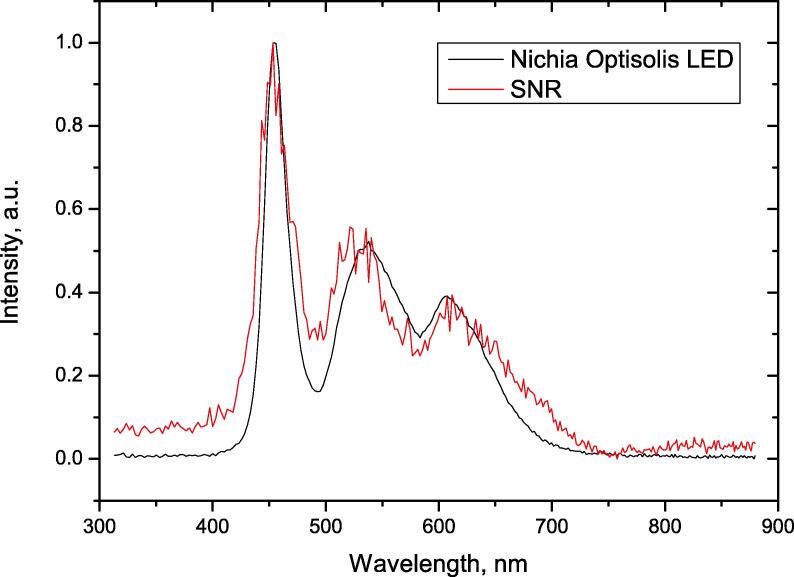
The normalized SNR spectrum and the spectrum of the light source LED (Nichia Optisolis). SNR value for peak at 540 nm is approximately 19 dB.

**Fig. 7:**
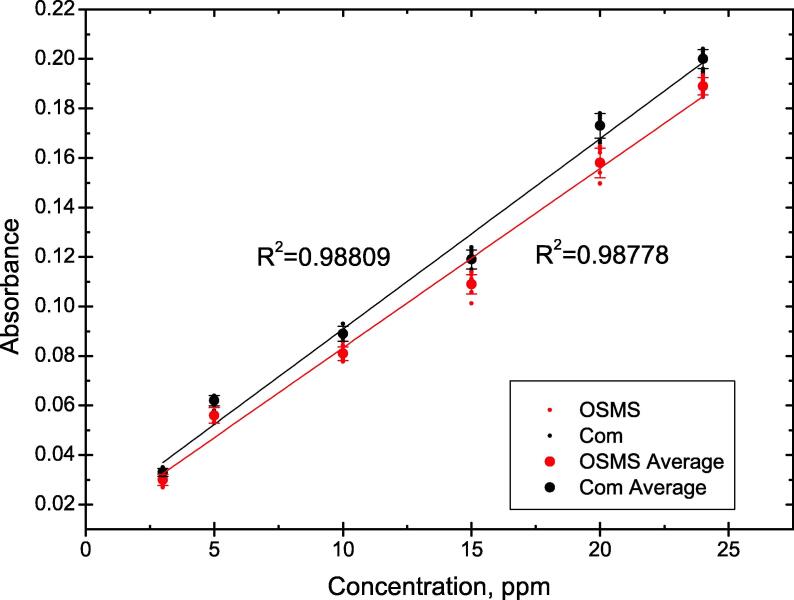
Vitamin B_12_ absorbance measurements and R^2^ values for the miniature spectrophotometer OSMS and the commercial *Com*. Average values of all measurements are represented (n = 9).

**Fig. 8:**
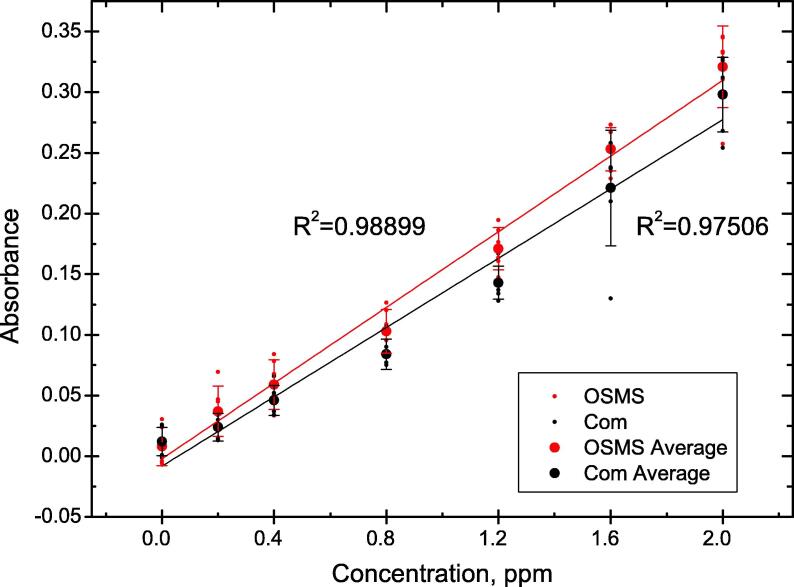
Phosphate absorbance measurements and R^2^ values for the open-source miniature spectrophotometer OSMS and the commercial one *Com*.

The 3D model was created in Autodesk Fusion 360 software. Overall dimensions of the assembled device are 90 × 85 × 58 mm (W × D × H). The case is assembled of three different parts main body, lid and top cap (lid for the cuvette) (Fig. 3a). Main body: allows simple assembly, three different spaces for LED diodes for future upgrades (Fig. 3-4), mounting point for the spectrometer unit (Fig. 3-6) as well as additional features: switch mount (Fig. 3-7), fixture hole (Fig. 3-8) and cuvette holding elements (Fig. 3-5). The lid contains holes for screws and light-tight mount for top cap an essential element for background-free measurement.When placed on a flat surface and the top cap lid is mounted, there is no significant amounts of light entering the measuring device.

For prototyping purposes, the case is optimized for fused deposition modeling (FDM) 3D printing minimal amount of supports is needed (only for holes and switch mount). All three parts of the design are printed separately. Material choice any thermoplastic filament. For better results and to lower the reflectivity of the internal surfaces a black color is desirable. Layer height 0.2 mm or lower, print time around 10 h depending on the precision and printer. Minimal infill (20%) is acceptable. Apart from the top cap, the design does not contain any moving components.

### 3.3 Software and the application

The device can be controlled in two ways. For simple and portable measurements, an Android application has been created (see Fig. 4), which offers the user to either acquire the whole absorption spectrum with the option to send it via e-mail for a more detailed analysis (Fig. 4a) or a specific wavelength can be set and the absorption value will be displayed on the screen (Fig. 4b).

The Python-based software for interfacing with a computer via a USB port offers the same functionality as the Android application with the additional options to automatically record and observe absorption data over time, set an extended accumulation time and export the data directly to the computer ((Fig. 4c).

### 3.4 Design files summary

**Table:** 

Design filename	File type	Open source license	Location of the file
Schematic	figure	GNU General Public License (GPL) 3.0	available athttps://doi.org/10.17605/OSF.IO/RBFSE
Android mobile application	.apk file	GNU General Public License (GPL) 3.0	available athttps://doi.org/10.17605/OSF.IO/RBFSE
MIT App Inventor project file	.aia file	GNU General Public License (GPL) 3.0	available athttps://doi.org/10.17605/OSF.IO/RBFSE
Python executable	.exe file	GNU General Public License (GPL) 3.0	available athttps://doi.org/10.17605/OSF.IO/RBFSE
Python source code	.py file	GNU General Public License (GPL) 3.0	available athttps://doi.org/10.17605/OSF.IO/RBFSE
3D design files	.stl file	GNU General Public License (GPL) 3.0	available athttps://doi.org/10.17605/OSF.IO/RBFSE

*Schematic:* The electronic schematic of the device with all the necessary components and their connections.

*Android mobile application:* The ready-to-install Android application file containing the application shown in Fig. 4.

*MIT App Inventor project file:* In order to enable the customization of the application for a wider range of users it was created using the MIT App Inventor. This is the project file which can be easily edited once opened via the MIT App Inventor.

*Python executable:* The executable file for desktop use. Offers accumulation functions as well as a continuous mode of measurements and file saving.

*Python source code:* The code behind the Python executable. Users can create changes here if need for customization arises.

*3D design files:* These are the 3D design files we used for our final container and lid printing.

## 4 Bill of materials

**Table:** 

Designator	Component	Number	Cost per unit currency	Total cost	Source of materials	Material type
U1	Arduino Microcontroller	1	20	20	Arduino	Electronics
U2	C12880MA Spectrometer	1	190	190	Hamamatsu	Electronics
U3	Bluetooth module HC-05	1	7.5	7.5	Amazon	Electronics
L1	LED Nichia Optisolis	1	2	2	Nichia Optisolis	Electronics
R1	Resistor 1 k	1	0.02	0.02	Local hardware store	Electronics
R2	Resistor 2.2 k	1	0.02	0.02	Local hardware store	Electronics
R3	Resistor 4.7 k	1	0.02	0.02	Local hardware store	Electronics
S1	Switch	1	0.10	0.10	Local hardware store	Electronics
PS1	9 V battery	1	2	2	Local hardware store	Electronics
Container	3D printed container	1	1.10	1.10	Fillamentum	3D printing
Lid	3D printed lid	1	0.4	0.4	Fillamentum	3D printing

## 5 Build instructions

The electronic parts should be soldered together as was shown in Fig. 2. Mind that the spectrometer C12880MA as well as the light source LED need to have longer wires since they are a bit farther away from the micro-controller. The 3D design was purposefully built more robust as a first prototype and could be made smaller if mounted on a PCB.

## 6 Operation instructions

### 6.1 To use the device without making changes

1. Assemble the device.
2. Upload Arduino code to your Arduino microcontroller.
3. Install Android app or run the Python software.

### 6.2 To obtain an absorption spectrum

1. Connect to Bluetooth if using app (you will need to pair your HC-05 module with your phone first)
2. Measure the baseline (the spectrum of the LED without the sample in).
3. Insert sample.
4. Choose either “Single” or “Continuous” measurement in Desktop mode or “Capture” in the Android app.
5. Repeat from step 2 for additional samples (however it is recommended to re-measure the baseline every once in a while).

Notes:

• Accumulation and continuous measurements are only possible in Desktop mode
• When changing accumulation time, make sure to measure the baseline again.

## 7 Validation and characterization

### 7.1 Characteristics of the spectrometer

To assess the quality of the main component of the system – the spectrometer chip, we determined the parameters that are frequently applied to commercial spectrometers in order to gauge their performance. Wavelength accuracy, dynamic range and signal-to-noise ratios were determined and are described below.

#### 7.1.1 Wavelength accuracy tests

The ready-built C12880MA spectrometer does not offer any calibration features and only comes with factory set wavelength positions for each pixel of the CMOS linear image sensor (that serves for accumulating the incoming light); therefore testing wavelength accuracy of the spectrometer itself was a key element in assessing the quality of future measurements. The accuracy was determined by measuring the emission spectra of an RGB LED with each color component turned on separately (Fig. 5) using the C12880MA spectrometer and a commercial Andor Shamrock B-303i spectrograph coupled with an Andor DU-401A-BV CCD camera. Slit width was kept the same (50 μm) for both spectrometers in order to acquire comparable results. The result is an average difference in the maximum emission wavelengths of 1.04 nm as compared to the Andor results. The shown data has not been corrected.

As the difference (step) between adjacent pixels of the CMOS sensor (for the OSMS) for R and G peaks are 2.09 nm, 2.34 nm (the pixel to wavelength conversion ir not linear), and the wavelength differences in maxima are less than half of that, it can be deduced that the maxima of the OSMS are as close to those of the commercial system as hardware limitations allow and cannot be viewed as real differences in the peak positions. For peak B, the difference between adjacent pixels is 2.2 nm and the difference between the OSMS and commercial system maxima is 1.3 nm, showing a bias towards the longer wavelengths that is not explained by adjacent pixel differences of the OSMS, however, it is also explained if the adjacent pixel step of the commercial system are also taken into account (0.55 nm).

#### 7.1.2 Signal-to-noise ratio and dynamic range

The dynamic range is a measure of the intensity resolution of a spectrometer and was 10 bits divided by the average of standard deviations for 50 measurements of dark noise with an exposure time of 200 μs.

$$\mathrm{Dynamic}\;\mathrm{range}=\frac{2^{10}-1}{{\mathrm{RMS}}_{\mathrm{dark}\;\mathrm{noise}}}=\frac{1023}{11.5}=89\;\text{dB}$$

The signal-to-noise ratio varies depending on wavelength and the chosen light source accordingly as can be seen in Fig. 6 (normalized data). The signal-to-noise ratio was calculated as the average value of 50 measurements divided by the standard deviation of the same measurements, using an exposure time of 200 μs. For the signal at 540 nm (light source supply power kept constant, intensity at 550 nm resulting in about half maximum) the SNR value was 19 dB showing that the obtained spectra are reliable enough for medium quality measurements.

$$\mathrm{SNR}=\frac{\mathrm{Signal}}{{\mathrm{RMS}}_{\mathrm{noise}}}=\frac{496.8}{6.4}=77.6=19\;\text{dB}$$

### 7.2 Comparison with commercial spectrophotometers

To test the OSMS in practical applications, three studies were performed and altogether additional 3 commercial spectrometers of various quality (price range) were used. For vitamin B_12_ and phosphate measurements the commercial spectrophotometer Shimadzu UVMini1240 was used (with a wavelength range from 190 nm to 1100 nm and a spectral bandwidth of 5 nm [16]. A bench-top photo-filter colorimeter (WPA colourvawe CO7500, filter bandwith 40 nm) and a high-quality (TECAN Infinite M200 PRO) (with a wavelength range from 230 nm to 1000 nm and a spectral bandwidth of <9 nm for >315 nm) were used for horseradish peroxidase activity measurements. The absorbance values presented are unmodified absorbance values obtained for the samples in a 10 mm × 10 mm cuvette. LOQ and LOD were calculated by the following formulas:

$$\mathrm{LOQ}=3.3\frac{S_{d}}{b}$$

$$\mathrm{LOD}=10\frac{S_{d}}{b}$$

where S_d_ is the standard deviation of the ordinate intercept and b is the slope of the regression line.

#### 7.2.1 Vitamin B_12_

The first set of absorption measurements were performed for various concentrations of vitamin B_12_, starting at 3 ppm and going up to 24 ppm. Vitamin B_12_ was chosen as the model analyte as it is water soluble and of high importance to the health of a human being [19], [12], making the measurements both simple and of practical use. Absorbance values were registered at 550 nm.

The obtained results show linear dependence (Fig. 7) between the concentration of B_12_ and the absorbance values in the range of concentrations tested. Similar R^2^ values for both the OSMS and the commercial bench-top spectrophotometer are seen. The tests were repeated on 3 different days (n = 3), performing 3 replicate measurements each day (n = 3) in order to determine the inter-day and the intra-day precision, respectively. The relative standard deviation can be seen in Table 1 for day to day measurements (inter-day precision) and the series measurements (intra-day precision). The results show slight differences in day to day measurements for both spectrophotometers, with a maximum RSD value of 6.60% obtained for the OSMS at the lowest concentration in comparison with a maximun RSD of 4.76% obtained for the bench-top commercial spectrophotometer (also at the lowest concentration). RSD values for the intra-day precision of the OSMS were found to be in the range of 3.78% and 0.82% in comparison with the RSD range of 3.33% and 0.70% found for the bench-top commercial spectrophotometer. Same LODs and very similar LOQs were obtained with both spectrophotometers.

**Table 1:** Absorbance values and calculated RSD, R^2^, LOD and LOQ for vitamin B_12_ concentrations in ranges from 3 ppm to 24 ppm – OSMS is the miniature spectrophotometer and *Com* is the commercial one.

			Inter-day (n = 3)		Intra-day (n = 3)	
	Absorbance		RSD, %		RSD, %	
	OSMS	Com	OSMS	Com	OSMS	Com
3 ppm	0.030	0.033	6.60	4.76	3.78	3.33
5 ppm	0.056	0.062	2.85	3.45	1.40	1.68
10 ppm	0.081	0.089	3.14	3.48	0.82	1.43
15 ppm	0.109	0.119	2.80	3.47	2.17	1.05
20 ppm	0.158	0.173	4.00	3.15	1.67	0.91
24 ppm	0.189	0.200	1.90	2.07	1.59	0.70
Average			3.55	3.39	1.90	1.52
R^2^	0.988	0.989				
LOD	2.7	2.7				
LOQ	8.3	8.2				

#### 7.2.2 Phosphate

Another set of measurements was performed for samples containing different levels of phosphate (up to 2 ppm) [9]. Phosphates are an essential nutrient and are most commonly used as a fertiliser, however, the unsystematic application of phosphates leads to the necessity of monitoring the amounts of phosphates entering the water system. Excessive amounts of phosphates can directly lead to the overpopulation of some organisms (such as phytoplankton), which in turn would affect the ecosystem as a whole if left unsupervised [22], [13]. The concentration of phosphates in large water systems is constantly monitored by environmental health organizations across the world, however, smaller water reserves are often left unmonitored and can loose their biodiversity. Handheld portable devices such as the open-source miniature spectrophotometer could make the examinations easier and more routine, allowing for greater control over the health of the local ecosystems. The represented absorbance values have been measured at 700 nm.

Although the obtained results show linear dependence between absorbance and concentration in the range of concentrations tested (Fig. 8), the obtained regression lines showed poorer fit for the UVMini1240 spectrometer measurements. As with B_12_, the tests were performed on different days (n = 2) for determination of inter-day precision and the corresponding SD values can be seen in Table 2.

**Table 2:** Absorbance values and calculated RSD, R^2^, LOD and LOQ for phosphate concentrations (expressed as total phosphorous) in ranges from 0.2 ppm to 2 ppm – OSMS is the open-source miniature spectrophotometer and *Com* is the commercial one.

			Inter-day (n = 2)		Intra-day (n = 3)	
	Absorbance		RSD, %		RSD, %	
	OSMS	Com	OSMS	Com	OSMS	Com
0 ppm	0.008	0.012	220.06	38.94	36.67	120.44
0.2 ppm	0.037	0.024	66.16	61.18	21.89	7.38
0.4 ppm	0.059	0.046	41.52	30.74	14.79	10.19
0.8 ppm	0.103	0.084	20.68	10.62	7.38	11.14
1.2 ppm	0.171	0.143	11.79	4.30	5.20	8.49
1.6 ppm	0.253	0.221	0.01	18.13	7.70	16.75
2 ppm	0.321	0.298	8.95	11.56	7.59	5.47
Average			52.74	25.07	14.46	25.69
R^2^	0.989	0.975				
LOD	0.2	0.3				
LOQ	0.5	0.8				

The RSD values for the obtained data can vary greatly when measuring low (null) absorbances (up to 220.06% for the OSMS and 120.44% for the benchtop spectrophotometer). Such measurements partially reflect the noise of the systems. Here standard deviations for the null measurement were 0.016 and 0.012 for the OSMS and the bench-top spectrophotometer, respectively. Combined with the lower absorbance value for the OSMS, the RSD value becomes significantly higher. The average standard deviation for all samples for both spectrophotometers differs only by 0.0006. Not taking into account the 0 ppm measurements, the average RSD values between the OSMS and the benchtop spectrophotometer differ by only 1–2% for both intra and inter-day measurements. RSD values for the intra-day measurements were found to be in the range of 5.20% to 21.89% for the OSMS and 5.47% to 25.69% for the commercial spectrophotometer. The OSMS showed a slightly lower LOD and LOQ.

#### 7.2.3 Horseradish peroxidase

Horseradish peroxidase (HRP) is an active and robust enzyme widely used as a reporter for coupled biochemical or immunological reactions [11], [10]. Many electron donors are used as substrates in peroxidase reactions, some of them changing their colour when donating an electron to an OH ion. 3,3,5,5 tetramethylbenzidine (TMB) is one of typical substrates used in HRP coupled reactions. When oxidised, the substrate changes its colour from colourless to torquise. In order to test the performance of the OSMS in determining HRP coupled reactions, spectrophotometric measurements of the reaction product (oxidised TMB) were performed. Low concentrations of H_2_O_2_ in the excess of enzyme and TMB were prepared to ensure that all H_2_O_2_ was oxidised to O_2_ and H_2_O and the blue colour present in the cuvette was directly proportional to the H_2_O_2_ concentration. The absorption of oxidised TMB was measured at 600 nm. Two different commercial spectrophotometers were used for comparison a simple bench-top WPA colourwave CO7500 (*Com1*) and the monochomator based spectrophotometer Infinite M200 PRO (*Com2*).

**Fig. 9:**
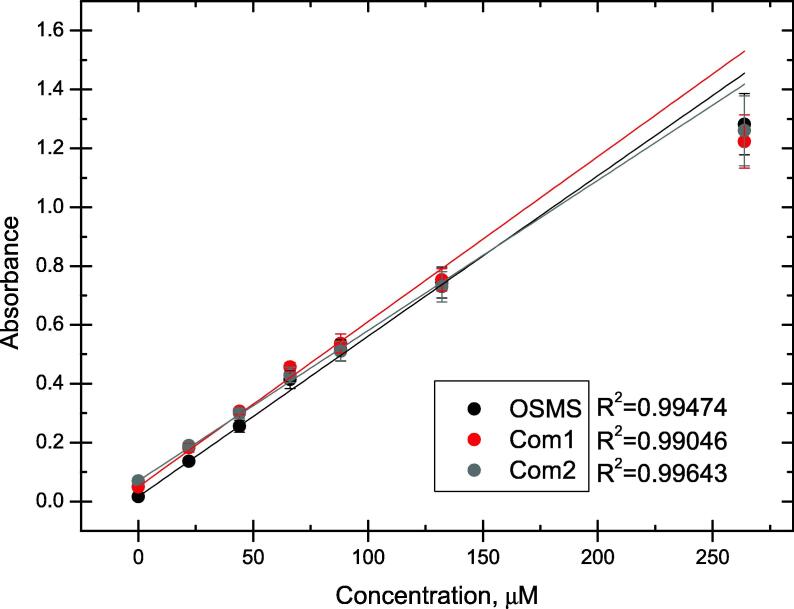
Hydrogen peroxide absorbance measurements and R^2^ values for the open-source miniature spectrophotometer OSMS and two commercial spectrophotometers – the low-cost (photo filter) *Com1* and the high-cost (monochromator) *Com2*.

The obtained results show linear dependency for concentrations in the range from 0 μM to 132 μM, with the linear region starting to diverge at 264 μM (Fig. 9). Despite the deviation from linearity, all of the spectrophotometers showed an R^2^ above 0.99 with R^2^ for the OSMS being higher than that of *Com1* and lower than R^2^ of *Com2*. Three replicate measurements were performed to obtain intra-day standard deviation values (n = 3). RSD values are found to be in ranges from 6.99% to 21.07% for the OSMS, 0.00% to 8.33% for the bench-top photo filter colorimeter and 3.09% to 9.43% for the monochromator based spectrophotometer (Table 3). Similar to phosphate measurements, the rather high RSD value of 21.07% is explained by the absorption value being lower with standard deviations on average being a similar 0.037, 0.029 and 0.036 for the OSMS, *Com1* and *Com2* respectively. LOD and LOQ were also significantly higher for the OSMS due to the same bias toward lower absorption values in general than for the commercial spectrophotometers.

**Table 3:** Absorbance values and calculated RSD, R^2^, LOD and LOQ for hydrogen peroxide concentrations in ranges from 22 μM to 264 μM – OSMS is the open-source miniature spectrophotometer and *Com1* and *Com2* are correspondingly the low-cost and high-cost commercial spectrophotometers.

	Absorbance			RSD, %		
	OSMS	Com1	Com2	OSMS	Com1	Com2
0 μM	0.016	0.050	0.066	21.07	0.00	3.09
22 μM	0.137	0.183	0.189	9.25	8.33	5.14
44 μM	0.256	0.307	0.296	7.90	3.77	5.81
66 μM	0.414	0.457	0.433	7.15	3.34	5.92
88 μM	0.514	0.537	0.515	6.99	5.99	6.40
132 μM	0.744	0.753	0.727	7.11	5.03	7.11
264 μM	1.282	1.223	1.260	8.10	7.42	9.43
Average				9.65	4.84	6.13
R^2^	0.995	0.990	0.996			
LOD	1.9	1.1	1.0			
LOQ	5.9	3.5	3.2			

## Declaration of Competing Interest

The authors declare that they have no known competing financial interests or personal relationships that could have appeared to influence the work reported in this paper.
